# Carbon based sensors for air quality monitoring networks; middle east perspective

**DOI:** 10.3389/fchem.2024.1391409

**Published:** 2024-05-20

**Authors:** Imran Shahid, M. Imran Shahzad, Ersin Tutsak, Mohamed M. K. Mahfouz, Maryam S. Al Adba, Saddam A. Abbasi, Hassaan Anwer Rathore, Zunaira Asif, Zhi Chen

**Affiliations:** ^1^ Environmental Science Centre, Qatar University, Doha, Qatar; ^2^ Department of Meteorology, COMSATS University Islamabad, Islamabad, Pakistan; ^3^ Department of Statistics, College of Arts and Science, Qatar University, Doha, Qatar; ^4^ College of Pharmacy, QU Health, Qatar University, Doha, Qatar; ^5^ Department of Engineering, University of New Brunswick, Saint John, NB, Canada; ^6^ Department of Building, Civil and Environmental Engineering, Concordia University, Montreal, QC, Canada

**Keywords:** carbon nanotubes, nanomaterials, air quality, sensors, IoT sensors network

## Abstract

IoT-based Sensors networks play a pivotal role in improving air quality monitoring in the Middle East. They provide real-time data, enabling precise tracking of pollution trends, informed decision-making, and increased public awareness. Air quality and dust pollution in the Middle East region may leads to various health issues, particularly among vulnerable populations. IoT-based Sensors networks help mitigate health risks by offering timely and accurate air quality data. Air pollution affects not only human health but also the region’s ecosystems and contributes to climate change. The economic implications of deteriorated air quality include healthcare costs and decreased productivity, underscore the need for effective monitoring and mitigation. IoT-based data can guide policymakers to align with Sustainable Development Goals (SDGs) related to health, clean water, and climate action. The conventional monitor based standard air quality instruments provide limited spatial coverage so there is strong need to continue research integrated with low-cost sensor technologies to make air quality monitoring more accessible, even in resource-constrained regions. IoT-based Sensors networks monitoring helps in understanding these environmental impacts. Among these IoT-based Sensors networks, sensors are of vital importance. With the evolution of sensors technologies, different types of sensors materials are available. Among this carbon based sensors are widely used for air quality monitoring. Carbon nanomaterial-based sensors (CNS) and carbon nanotubes (CNTs) as adsorbents exhibit unique capabilities in the measurement of air pollutants. These sensors are used to detect gaseous pollutants that includes oxides of nitrogen and Sulphur, and ozone, and volatile organic compounds (VOCs). This study provides comprehensive review of integration of carbon nanomaterials based sensors in IoT based network for better air quality monitoring and exploring the potential of machine learning and artificial intelligence for advanced data analysis, pollution source identification, integration of satellite and ground-based networks and future forecasting to design effective mitigation strategies. By prioritizing these recommendations, the Middle East and other regions, can further leverage IoT-based systems to improve air quality monitoring, safeguard public health, protect the environment, and contribute to sustainable development in the region.

## 1 Introduction

The Middle East, a region known for its historical richness, cultural diversity, and economic significance, is also grappling with a pressing concern that affects the lives of millions of its inhabitants - poor air quality. As the region undergoes rapid urbanization, industrialization, and population growth, the importance of air quality monitoring in the Middle East has never been more evident. The Middle East is the conjunction of expanding urbanization, industrialization and agriculture along with inherited desertification. This provides a homegrown persistent source of air pollution affecting quality of life of residence by aggravating the health issues pertaining to respiratory functions irrespective of gender and age. Fine particulate matter (PM2.5) and pollutants such as nitrogen dioxide (NO2), Ozone (O3) and sulfur dioxide (SO2) pose serious health risks, particularly in densely populated urban areas (Dockery and Pope, 1994).

Vulnerable groups, such as children, the elderly, and individuals with preexisting health conditions, are at higher risk. Air pollution exacerbates respiratory ailments, leading to increased hospitalizations and premature deaths, burdening healthcare systems and affecting the overall wellbeing of communities (Kampa and Castanas, 2008). Beyond human health, air pollution takes a toll on the region’s unique ecosystems. It harms vegetation, disrupts fragile desert ecosystems, and affects biodiversity. Dust storms, a recurring phenomenon in the Middle East, carry pollutants that can travel vast distances, impacting regions far from their source (Awadh, 2023). Air pollutants like black carbon contribute to climate change by accelerating the melting of snow and ice in surrounding regions. As the region faces increasing temperatures and shifting climate patterns, air quality monitoring becomes essential for understanding and mitigating these changes (Lelieveld et al., 2015). Some common gaseous pollutants and PM alongwith their sources have been given in Table 1.

**TABLE 1:** Common pollutants and their sources.

Pollutants	Main sources
NOx (NO, NO2, N2O)	Burning of fuel in automobiles and industry
CO	Fossil fuel Combustion in transport and power generation
CO2	Fossil fuel, cement construction and vehicles, livestock
SO2	Fossil fuel Combustion, power generation
VOC	Plants, chemical industry, cleaners, disinfectants
Particulate matter (PM10, PM2.5)	Vehicles exhaust, construction sites, open garbage, resuspended dust

Monitoring air quality in the Middle East is not merely a response to a pressing problem; it is a beacon of hope for the region’s future. The deployment of advanced air quality monitoring networks, often integrated with Internet of Things (IoT) technology, offers several advantages (Alghamdi et al., 2015).

IoT-based sensor networks provide real-time data on air quality, enabling authorities to respond swiftly to pollution events and protect public health. Reliable air quality data informs policy and regulatory decisions, supporting efforts to reduce emissions and improve air quality standards. Accessible air quality information empowers citizens to make informed choices about outdoor activities, reducing personal exposure to pollution. Data collected from monitoring networks drive research on air quality and its effects, fostering innovation in pollution control technologies and sustainable urban planning.

## 2 Air quality in the middle east

The Middle East faces significant air quality challenges driven by rapid urbanization, industrialization, and natural factors. Air quality issues are exacerbated by unique regional characteristics, including arid climates and increasing dust storms, which introduce additional pollutants into the atmosphere (Goudie, 2014). Dust and fine particulate matter (PM10 and PM2.5) are major pollutants in the Middle East. Sources include natural dust storms, construction activities, and industrial emissions (Nisbet et al., 2019). Traffic emissions, industrial processes, and energy production contribute to elevated NO2 levels in urban areas (Duncan et al., 2016). SO2 emissions arise from industrial activities, including oil refining and power generation (Roy and Sardar, 2015). VOCs are released from transportation, petrochemical industries, and natural sources. They contribute to ozone formation and smog (Siddiqua et al., 2022). Ozone is a secondary pollutant formed through photochemical reactions involving VOCs, NOx, and sunlight.

IoT-based sensor networks offer crucial advantages in addressing air quality challenges in the Middle East. IoT sensors provide real-time data, enabling timely responses to pollution events, especially dust storms (Zheng et al., 2018). Distributed sensor networks offer comprehensive spatial coverage, essential for understanding pollution patterns across cities and regions (Saini et al., 2021). IoT sensors assist in identifying pollution sources, facilitating targeted mitigation efforts. Accessible air quality information empowers citizens to protect their health and advocate for pollution control measures. Reliable IoT-based data informs evidence-based policies and regulatory decisions, critical for improving air quality standards (Saini et al., 2021)

## 3 Sensors used for air quality monitoring

An air quality monitoring system can be considered comprehensive if it hosts a variety of sensors to monitor the air as well as weather. There is a large list of available options, but few are considered very much indispensable.

Gas sensors detect specific pollutants (e.g., NO2, CO, VOCs) through chemical reactions that change electrical conductivity, resistivity, or optical properties (Zheng et al., 2018). Gas sensors offer high precision and accuracy for targeted gases but may require calibration to maintain accuracy over time. However, regular maintenance and calibration is inevitable for quality assurance (Karagulian et al., 2019). Ozone (O3) is measured through fluctuations in electrical signals due to reactions triggered electrochemically (Gao et al., 2012). Like other sensors, this also demands frequent calibration and maintenance for ensuring quality of data. Temperature and Humidity sensors use thermistors and capacitors to convert changes in electrical flow into temperature and humidity values (Zheng et al., 2018).

Particulate Matter (PM) sensors measure the concentration of airborne particles by capturing and counting particles using light scattering or light blocking techniques (Gao et al., 2015). PM sensors exhibit good accuracy for larger particles (PM10) but may have reduced accuracy for fine particles (PM2.5) due to variability in particle composition (Kelly et al., 2017). Regular maintenance, including cleaning of sensor components and calibration, is necessary to ensure accuracy.

### 3.1 Carbon based sensors

**FIGURE 1:**
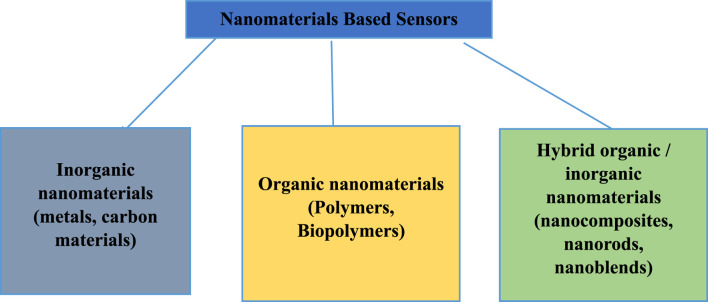
Nanomaterial based sensors types.

Advancement in nanotechnology and nanomaterials production has given vital solution in almost every field of life. Among these, development of smart sensor and biosensor has provided the alternatives for conventionally used analyzers and techniques for environmental pollutants sensing that has low sensitivity, high power consumption, and high cost (Alwin David et al., 2022; Gulati et al., 2022). The nanomaterial-based sensors are low cost, handy and have high sensitivity. The chemical sensor comprises of a transducer and an active layer of material that translates chemical information into electronic signal, such as change in frequency, voltage or current. There are different types of gas sensor based on their applications, measurement principle and performance. These are semiconductor, electrochemical, acoustic, catalytic and optical gas sensors. Nanomaterials based chemical sensors for detecting air pollutants are widely used (Gabal et al., 2020; Gulati et al., 2022). These sensors are lower cost monitoring devices that can efficiently measure gases and particle in ambient air. Due to large surface area-to-volume ratios their performance and sensitivity is high and these sensors are able to detect minute quantity of pollutants in air. Nanomaterial receptors include organic materials (polymers, bio-polymers, etc.), inorganic materials (carbon and metals) and hybrid materials. Among these carbon nanomaterials (CNM) have shown significant progress due to their thermal, optical, mechanical and electrochemical properties (Ibrahim et al., 2016; Zhang et al., 2019; Gabal et al., 2020; Dhall et al., 2021). These materials are carbon nanotubes (CNT), nanohorns, graphenes, fullerenes and carbon dots widely used in sensing technologies. Carbon based nanostructures like CNTs and graphene have capacity to detect extremely low concentrations of gases. The schematic diagram of the carbon nanomaterials based sensors if in Figure 1.

#### 3.1.1 Carbon nanotubes (CNTs)

Graphene are extensively used in sensor manufacturing due to excellent performance in detecting variety of gaseous pollutants like VOCs, CO, CH4, NO2, NH3, H2S and SO2 (Mittal and Kumar 2014; Meyyappan, 2016; Schroeder et al., 2019; Zhang et al., 2019; Abdel-Karim et al., 2020; Gabal et al., 2020; Gulati et al., 2022). The structural characteristics of CNTs such as surface area, affinity of pollutants, high porosity, low density and functionalization made them more efficient and reliable. There are different types of CNTs based on their structure and applications.

**FIGURE 2:**
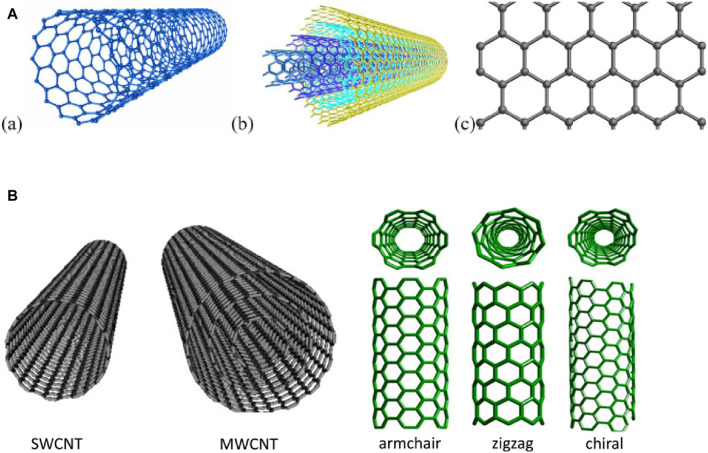
**(A)** SWCNT, **(B)** MWCNT and **(C)** graphene (Ouyang, 2019) **(B)**. Different types of CNT in grey are models of either single-walled or multi-walled CNTs and in green are the various forms of SWCNTs (Madani et al., 2013).

The carbon atoms arranged in cylindrical tubes with diameters ranging from 1–100 nm, are classified as single-walled (SWCNTs) or multi-walled (MWCNTs) (Sinha and Yeow, 2005; Madani et al., 2013). These hexagonal rings regulate their properties and are synthesized using different methods. The commonly used methods are laser ablation, chemical vapor deposition (CVD) and arc discharge (Beg et al., 2010; Zhang et al., 2019). In the CNT based sensors, gases react at the surface of CNTs and changed sensor resistance that is measured as electrical signal. CNTs sensors provides high sensitivity at ambient temperatures. The combination of metal oxides and CNT further enhance the sensing capacity of the materials gases and these sensors can detect VOC in air. The structure of CNT and grephene is given in Figure 2.

#### 3.1.2 Quantum Dots (QDs)

Inorganic nanoparticles with low fluorescence emission bands. QDs are made of very small number of atoms with distinct energy spectrum. QDs can be used as optical transducers in gas sensors. The flexible wavelength has made QDs more suitable for multi-detection of different analytes (Galstyan, 2021; Alwin David et al., 2022; Kumar et al., 2022; Luo et al., 2022). The sensing behavior of QDs depends upon the structure, composition, and synthesis method. QDs based sensors provides better surface area and charge transfer as compared to conventional devices. Due to their unique structural properties and broad absorption spectra, QDs can be excited by a single energy source. The graphene quantum dots and CdSe, CdSe/ZnS, CdTe, CdTe/CdS, and ZnS based QDs are widely used in sensors (Zhang et al., 2017; Galstyan, 2021; Geng et al., 2021; Lee et al., 2022; Luo et al., 2022). Due to zero-dimensional semiconductor nature of QDs with distinctive electrical and optical properties, QDs are also called “artificial” atoms (Galstyan, 2021; Alwin David et al., 2022; Kumar et al., 2022; Lee et al., 2022). There are two methods for development of semiconductor QDs for chemical gas sensing applications, i.e., epitaxial and colloidal. The ligand formation of the analytes surfaces restrict its reactivity with gases, this process decreases it efficiency, but it can controlled by treating QDs surfaces. The graphene QDs with SnO2 show extraordinary performance and high sensitivity with low detection limit against different gases like NO, CO, CH3COCH3 (Sun et al., 2020; Chen et al., 2021) while QDs with PbS and PbSbS showed excellent behavior against CO (Kumar et al., 2022). There are large number of studies that used QDs some examples of these sensors are given below in Table 2.

**TABLE 2:** Quantum dot-based Sensors.

Nanostructure	Targeted contaminants	Reference
SnS, C(S,N)-WO3, MoS2/SnO2, ZnO-multilayer graphene, PbCdSe, Carbon/In2O3	NO2	Li et al., 2019; Chen et al., 2021; Geng et al., 2021; Kumar et al., 2022; Lee et al., 2022; Luo et al., 2022; Patel et al., 2022
TiO2/PbSnS	CO, NO2	Kumar et al. (2022)
N-Graphene QDs/SnO2, ZnO-SnO2	CH2O	Sun et al., 2020; Chen et al., 2021
ZnO	H2S	Zhang et al. (2017)

#### 3.1.3 Metal oxide nanoparticles sensors (MoS)

Among the other nanomaterial sensors for environmental monitoring and gas sensing technology metal oxide nanoparticles sensors are emerged as exceptional performance. These nanostructures are usually one-dimensional (1D) and revolutionized electrochemical sensing. Likewise, CNT and QDs, MoS high sensitivity and selectivity is due to their surface to volume ratio. MoS based on tin, titanium, iron, zinc and cerium oxides are widely used for gas sensing. These sensors also show extraordinary performance in detecting volatile organic compounds (VOC) present in the air (Zhao and Yang, 2003; Fu and Liao, 2012; Gulati et al., 2022). The titanium dioxide (TiO₂) nanoparticles are usually used in photocatalytic activities but in nano sensor system it has emerged as a vital constituent. In air pollutant detection Tin oxide (SnO₂) is widely used due to its high sensitivity at low temperatures and cost-effectiveness (Dascalu et al., 2018; Shinde et al., 2022). ZnO nanorods exhibit impressive sensitivity to NH₃ in a wide range of temperatures (Tonezzer, 2019; Lupan et al., 2022). Additionally, the molybdenum disulfide (MoS2) modified with reduced graphene oxide (rGO) can also improve the detection sensitivity and selectivity in gas sensors (Niu et al., 2015; Sangeetha and Madhan, 2020; Chen et al., 2021; Rawat et al., 2021; Luo et al., 2022; Liu et al., 2023) provided a metal oxide based sensors framework for air quality monitoring. Summary of some commonly used metal oxide sensors have been given in Table 3.

**TABLE 3:** Metal oxide-based sensors.

Nanostructure	Targeted contaminants	Reference
rGO/Pd coated SnO2 film, WOx, WO3-graphene@Cu, Porous rod-like In2O3	NO2	Tonezzer, 2019; Dhall et al., 2021; Wang et al., 2021; Haiduk et al., 2022; Isaac et al., 2022; Li et al., 2022
ZnO:Eu nanowire, Comb-like ZnO	H2	Lupan et al. (2022)
Single SnO2 nanowire, ZnO/CuO	C3H6O, NH3, CO, C2H6O, H2, NO2, C7H8	Tonezzer, 2019; Shinde et al., 2022
Ni, Zn doped SnO2, WO3-graphene@Cu, Zn, Fe modified SnO2	CO	Dascalu et al., 2018; Zhou et al., 2018; Tonezzer, 2019; Haiduk et al., 2022
WO3-graphene@Cu	CO, NO2, C3H6O	Haiduk et al. (2022)

## 4 Air quality monitoring networks

**FIGURE 3:**
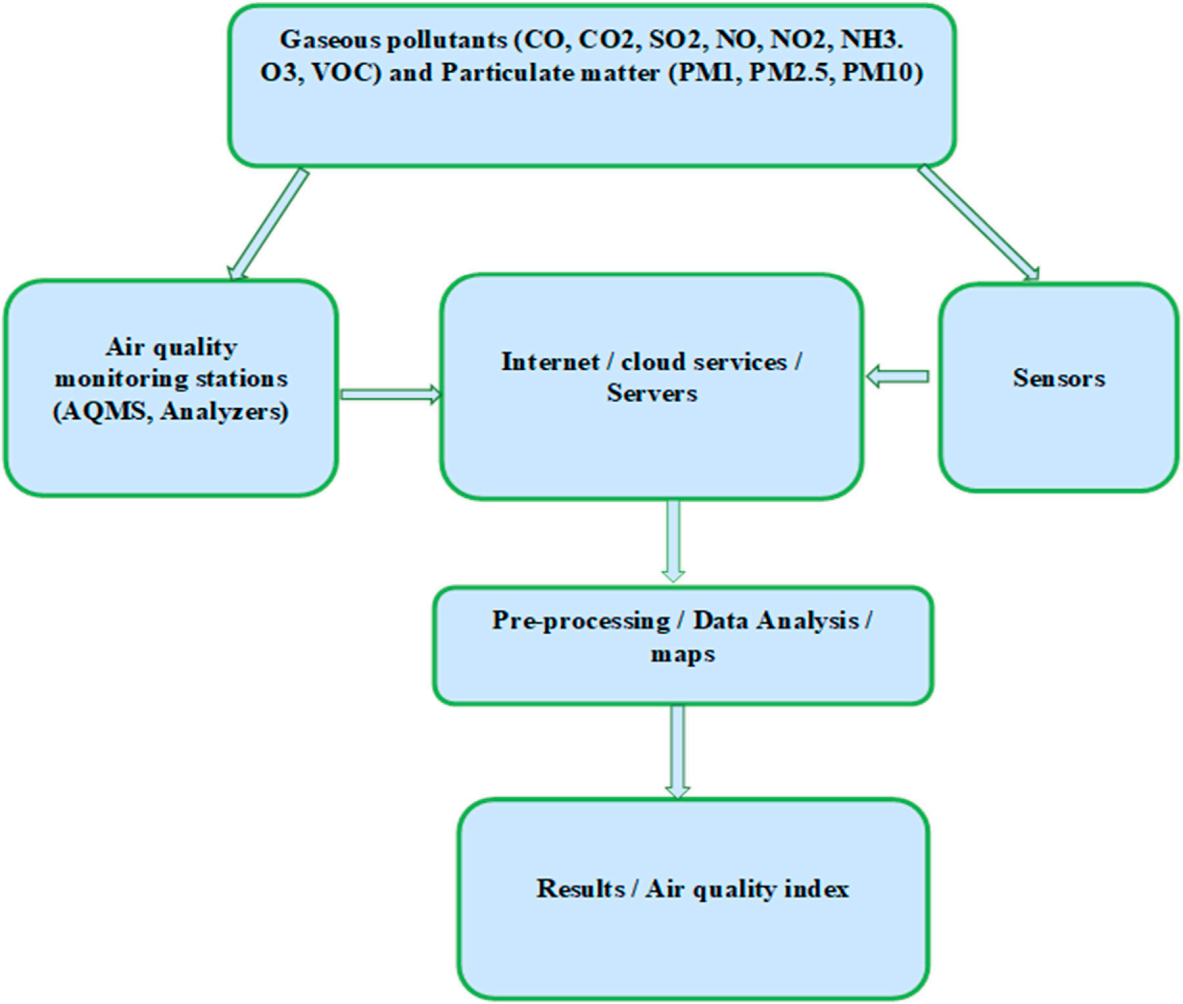
Integrated Air quality network design.

**FIGURE 4:**
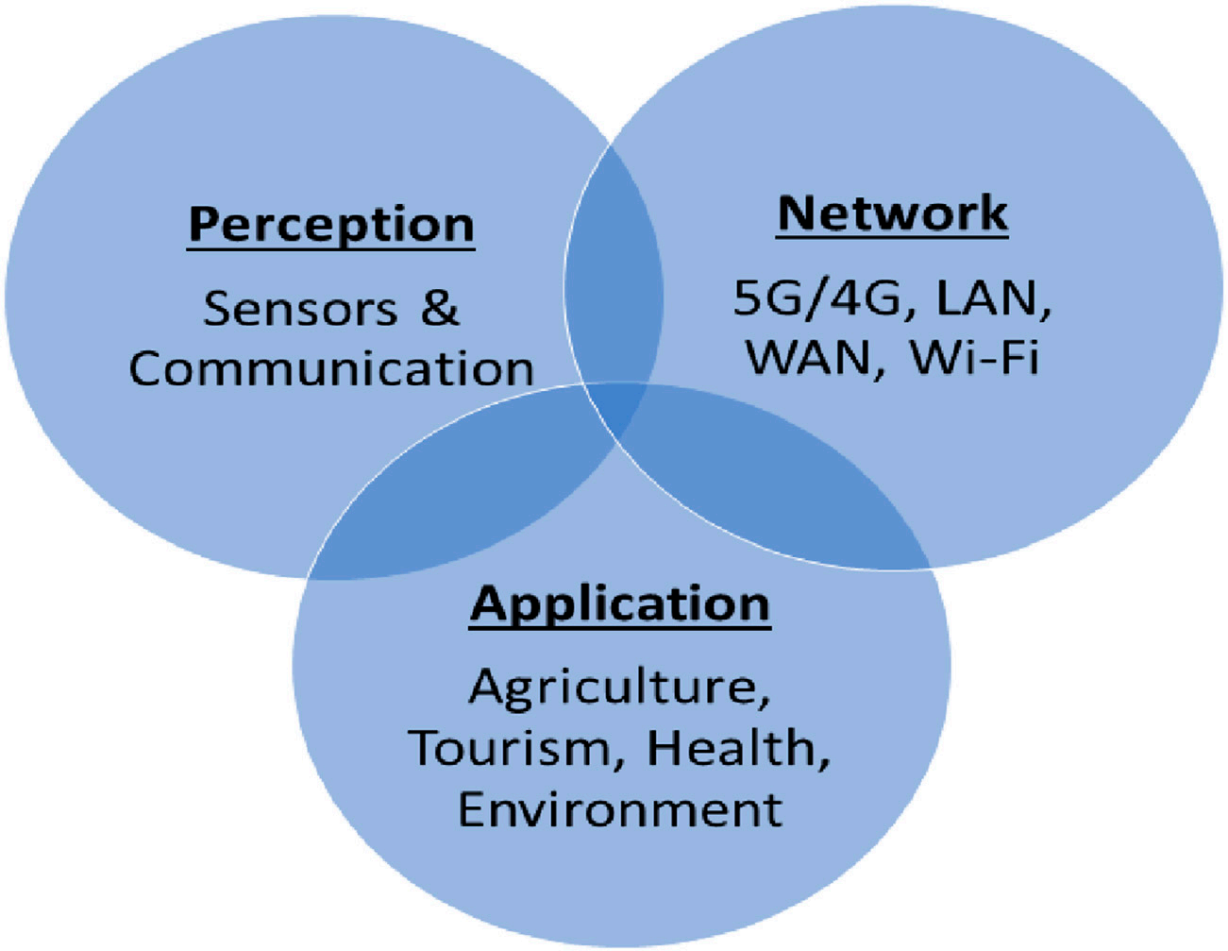
Summary of three tiers of an IoT based Air Quality monitoring network.

A large number of companies are designing and manufacturing sensors for Air quality monitoring using different materials. These sensors are categorized based on their performance, cost effectiveness as well as working conditions. A smart IAQ monitoring network works with on-the-go data transfer from sensors to server on cloud for subsequent IAQ processing and evaluation (Husain et al., 2007; Arar and Jung, 2022; Asghar et al., 2024). IoT based network also comprises of end equipment and communication exchange facilities. These components exchange information autonomously providing users with intellect, intense, and rapid response. A Schematic model of IoT based air quality network consists of user, cloud, internet, mobile network, sensors and data logger or hub/gateway (Figure 3). Although it is financially expensive to use cloud-based services, it can be the backbone for an efficient network dealing with big data analysis at operational level. This also can ensure security of the data with high level of possible encryption because end user interact with the network via variety of the devices such as smartphones, tablets, or laptops. These communication channels can be divided into three tiers of perception, network, and application (Figure 4). Perception tier is the core of the system consisting of air quality and weather sensors, and devices to record the information and send it into network (Gharaibeh et al., 2017). Network tier may consist of any communication protocol or network such cellular, LAN, WAN or wireless to efficiently disseminate the information retrieved from perception tier to user through application tier connecting various applications (Sai et al., 2019; Bousiotis et al., 2021; Buelvas et al., 2023).

### 4.1 IoT sensors network

IoT is extensively employed in air quality monitoring to gather real-time data from various sensors dispersed across urban areas and remote locations. These systems are instrumental in tracking key air pollutants, including particulate matter (PM), nitrogen dioxide (NO2), sulfur dioxide (SO2), volatile organic compounds (VOCs), and ozone (O3) (Saini et al., 2021).

IoT-based air quality monitoring systems offer several advantages over conventional networks. IoT sensors provide real-time, continuous monitoring of air quality, enabling rapid response to pollution events (Zheng et al., 2018). Distributed sensor networks offer comprehensive spatial coverage, facilitating a detailed understanding of pollution patterns across regions (Saini et al., 2021).

There have been examples of successful implementation of IoT based air quality monitoring networks in the world. China-Beijing’s comprehensive IoT-based air quality monitoring network includes over 35,000 sensors. It provides real-time data to citizens and authorities, enabling effective pollution control measures. London, UK runs the Breathe London project that utilizes IoT sensors to monitor air quality across the city. Data from over 100 fixed sensors and mobile monitoring units inform public health initiatives and policy decisions (Ma, 2021). Delhi - India’s air quality monitoring network relies on IoT sensors to track PM2.5 and PM10 levels. The data aids in issuing health advisories and implementing traffic control measures. Similarly, there are numerous examples of such IoT based networks to get inspired.

### 4.2 Data analysis and visualization

Collected IoT sensor data undergoes several stages of analysis and processing such as Preprocessing, Extraction, Modeling, and lastly Visualization. Raw data is cleaned, filtered, and transformed to remove noise and outliers, ensuring data quality (Khosravi et al., 2011). Relevant features are extracted from the data, which can include statistical measures, trends, or anomalies (Khosravi et al., 2011). Data is used to build models or algorithms that can predict future trends, detect anomalies, or provide insights (Chen et al., 2014). Data visualization plays a crucial role in making complex IoT data understandable and actionable. Line graphs, bar charts, and scatter plots are commonly used to visualize time-series data and relationships between variables (Hassan et al., 2020). Heat maps help represent data density and patterns, making it easier to identify hotspots or trends in spatial data. Mapping IoT data onto geographic information systems (GIS) helps visualize spatial trends and relationships.

Real-time reporting of IoT data is crucial for proactive and timely decision making. Real-time reporting enables immediate actions in response to critical events or anomalies, such as pollution spikes or security breaches (Bales et al., 2014). Real time reporting also brings in proactive maintenance allowing predictive maintenance, reducing downtime and maintenance costs for IoT devices and systems (Him et al., 2019). Most importantly real time reporting of an IoT network increases public awareness and empowers the public with up-to-the-minute information on air quality, traffic, and other factors that impact daily life.

## 5 Air quality monitoring sensors networks in Middle Eastern countries

Geopolitical and socioeconomic importance of Middle East merits the sustainable existence of IoT based air quality monitoring networks in the region to work synergistically for equally good air quality of the region. Regionally integrated air quality monitoring networks will serve several stakeholders related to departments ranging from educational, research, policy, health, environment, tourism and many more. There are already a few successful examples of such IoT based air quality networks in the Middle East (Table 4). These networks play a vital role in assessing and managing air quality in the Middle East, helping to protect public health and the environment while informing policy decisions and regulatory actions.

**TABLE 4:** Urban air quality management systems Middle East Regions.

Country	Air quality management process	Scale	Reference
Bahrain	Continuous monitoring was set up at four geographical locations in 1993 to monitor major air pollutants	City	Jassim and Coskuner, (2017)
Egypt	In 1998, an air quality monitoring network of 87 stations was established. 128 chimneys monitor emissions from the cement, fertilizer, and petrochemical industries	City	HOWES and ABDEL-REHIEM, (2012)
Jordan	Jordan Environmental Monitoring Network under the control of Ministry of Environment	National	Fezari et al. (2015)
Morocco	Air quality measures and automobile emission monitoring in large cities are conducted using aerosol and gas sampling, ambulant laboratories, and heavy metal analytical procedures	City	Chirmata et al. (2017)
Qatar	Qatar Air Quality Monitoring Network is controlled by the Qatar Ministry of Municipality and Environment with mandate to establish fixed and mobile air quality monitoring stations	City	Yaacoub et al. (2013)
Saudi Arabia	Saudi Environmental Monitoring Network (SEMN) is operated by Ministry of Environmental Protection in collaboration with Saudi Aramco with 10 environmental and 15 meteorological stations	City	Mofarrah and Husain, (2010)
UAE	Dubai Air Quality Monitoring Network (DAQMN) was established by Dubai Municipality in 2003 with 15 permanent and 2 mobile stations. Additionally, 46 air quality monitoring stations near cement plants	City	Omidvarborna et al. (2018)

Countries in the initial stages of implementing air quality networks at regional level must understand the responsibility and roles of the important stakeholders such as Government Agencies, Research Institutions, Environmental Organizations and public. Government agencies are often responsible for establishing and maintaining monitoring infrastructure, setting air quality standards, and regulating emissions. Research institutions contribute to data collection, analysis, and research on air quality, helping to improve monitoring techniques and policy recommendations (Hassan et al., 2020). Environmental organizations advocate for air quality improvements, raise public awareness, and sometimes operate independent monitoring stations (Sarigiannis et al., 2011). The role of the public is of the utmost importance to reach a sustainable clean environment reflected from their actions and awareness. Despite the importance of collaboration, several challenges hinder effective data sharing and cooperation in the Middle East which is linked to the unawareness or lack of understanding of the roles of stakeholders which usually leads to the limited accessibility to air quality data due to proprietary ownership, lack of standardized formats, and data restrictions by some stakeholders (Buelvas et al., 2023; Srishti et al., 2023). Data recorded by diverse sources often faces issues of interoperability when it converges in a network. Variability in data formats, measurement units, and quality control procedures among different monitoring stations hampers data integration and analysis (Din et al., 2019; Hassan et al., 2020). There is a lack of regulatory harmonization among the Middle Eastern countries due to several reasons. For instance, disparities in air quality regulations among Middle Eastern countries hinder regional cooperation and consistency in addressing cross-border pollution (Sarigiannis et al., 2011; Sarigiannis et al., 2017). Further, limited resources and funding for monitoring infrastructure and research impede the expansion and maintenance of monitoring networks.

The Middle East can consider Dubai Air Quality Monitoring Network (DAQMN) as an example to follow for establishing an air quality network embedded with IoT. DAQMN can be looked up as an air quality network covering various types of possible air pollution sources from urban, rural, industrial and natural. DAQMN is promoted as transparent source of information to both public and policymakers through real time dissemination of information through web and mobile platforms (Environment, 2024).

## 6 Effectiveness of IoT-based monitoring

IoT-based systems have brought significant advancements to air quality monitoring in the Middle East, leading to a deeper understanding of pollution trends, notable improvements, and positive changes in public awareness and policies. IoT-based systems provide real-time and precise data on air quality, allowing for the identification of pollution hotspots and the sources of contaminants (Him et al., 2019; Sai et al., 2019; Bousiotis et al., 2021; Saini et al., 2021; Buelvas et al., 2023; Asghar et al., 2024). Continuous monitoring has revealed seasonal and diurnal pollution patterns, aiding in the development of targeted mitigation strategies (Chen et al., 2015; Ammar et al., 2018; Din et al., 2019; Hassan et al., 2020; Saini et al., 2021; Saini et al., 2021; Buelvas et al., 2023). The data-driven approach has enabled governments to measure the effectiveness of pollution control measures and adapt policies accordingly.

Accessible air quality information through IoT platforms has empowered the Middle Eastern public to make informed decisions about outdoor activities and reduce personal exposure to pollution. Public pressure, driven by increased awareness, has catalyzed policy changes, leading to stricter emissions regulations, increased green initiatives, and support for cleaner transportation options (Zanella et al., 2014; Chen et al., 2015; Ammar et al., 2018; Čolaković and Hadžialić, 2018; Hassan et al., 2020; Saini et al., 2021; Kausar et al., 2023). IoT-based data has become integral to policy development, enabling evidence-based decision-making and fostering international cooperation in addressing air quality challenges (Sarigiannis et al., 2011; Sarigiannis et al., 2017).

## 7 Challenges and future directions

Implementing IoT-based systems for air quality monitoring in the Middle East faces several challenges such as infrastructure constraints, data privacy and security, and interoperability. Limited infrastructure in some areas may hinder the deployment of monitoring networks, particularly in remote or underdeveloped regions (Abomhara and Køien, 2014). Ensuring data privacy and security is crucial, given concerns related to data breaches and misuse of sensitive information (Miorandi et al., 2012). The lack of standardized protocols and data formats can hinder interoperability between different monitoring systems and devices (Al-Fuqaha et al., 2015). However, to overcome these challenges governments must invest in infrastructure development, including the expansion of wireless networks and power supply. This can facilitate the deployment of IoT-based systems in remote areas (Bales et al., 2014). To make this network sustainable, robust data encryption and access control mechanisms should be in place to protect sensitive information, addressing data privacy and security concerns (Zheng et al., 2018). Most importantly, a Regional Spatial Data Infrastructure (RSDI) or other data sharing protocols are necessary to capitalize the use of collected data from IoT network (Miorandi et al., 2012).

Use and development of low-cost air quality monitoring sensors should be encouraged at policy level to achieve high spatial and temporal coverage (Margaritis et al., 2021; Bousiotis et al., 2023; Buelvas et al., 2023; Nobell et al., 2023). Similarly, the use of artificial intelligence-based algorithms should be encouraged to process the collected data. This will enable real time availability of accurate reliable data for enhancing the scientific understanding of air pollution trends and sources for effective policy making (Chen et al., 2014).

## 8 Conclusion

An integrated air quality monitoring network based on Nanomaterial based sensors is an indispensable element for research, teaching, awareness and policy making to produce knowledgeable decisions in the vast interest of public of Middle East. Air quality in the Middle East is strongly impacted by dust. The following recommendations has been made based on this study.

➢ Integrated IoT-based sensor network systems with conventional air quality monitoring network will increase geospatial data coverage that will leads to better air quality management.
➢ The choice of sensor depends on factors such as the specific gases or particles to be detected, desired sensitivity and selectivity, environmental conditions, cost considerations, and integration requirements. Therefore, a comprehensive evaluation considering these factors is necessary to determine the most suitable sensor for a particular application.
➢ IoT-based monitoring will enhance understanding the impact of air quality of region’s ecosystems, environmental and climate change impacts.
➢ IoT-based data guides policy changes, aligning with Sustainable Development Goals (SDGs) related to health, clean water, and climate action.
➢ The use of new techniques like machine learning and artificial intelligence for advanced data analysis will improve pollution source identification and development of early warning system for extreme weather events like dust storms.
➢ The integration of satellite and UAV-based sensing technologies will complement ground-based networks and boost understanding of the pollution sources
➢ To tackle transboundary pollution sources within the region standardization of data formats and unified protocols are required to enhance data interoperability and collaboration.
➢ Public awareness and participation in air quality monitoring efforts is vital to foster responsible behaviors.

By prioritizing these recommendations, the Middle East can further leverage IoT-based systems to improve air quality monitoring, safeguard public health, protect the environment, and contribute to sustainable development in the region.
